# Hexadecanamide alleviates *Staphylococcus aureus*-induced mastitis in mice by inhibiting inflammatory responses and restoring blood-milk barrier integrity

**DOI:** 10.1371/journal.ppat.1011764

**Published:** 2023-11-10

**Authors:** Lijuan Bao, Hao Sun, Yihong Zhao, Lianjun Feng, Keyi Wu, Shan Shang, Jiawen Xu, Ruping Shan, Shiyu Duan, Min Qiu, Naisheng Zhang, Xiaoyu Hu, Caijun Zhao, Yunhe Fu

**Affiliations:** Department of Clinical Veterinary Medicine, College of Veterinary Medicine, Jilin University, Changchun, Jilin Province, China; University of Michigan, UNITED STATES

## Abstract

Subacute ruminal acidosis (SARA) has been demonstrated to promote the development of mastitis, one of the most serious diseases in dairy farming worldwide, but the underlying mechanism is unclear. Using untargeted metabolomics, we found hexadecanamide (HEX) was significantly reduced in rumen fluid and milk from cows with SARA-associated mastitis. Herein, we aimed to assess the protective role of HEX in *Staphylococcus aureus* (*S*. *aureus*)- and SARA-induced mastitis and the underlying mechanism. We showed that HEX ameliorated *S*. *aureus*-induced mastitis in mice, which was related to the suppression of mammary inflammatory responses and repair of the blood-milk barrier. *In vitro*, HEX depressed *S*. *aureus*-induced activation of the NF-κB pathway and improved barrier integrity in mouse mammary epithelial cells (MMECs). In detail, HEX activated PPARα, which upregulated SIRT1 and subsequently inhibited NF-κB activation and inflammatory responses. In addition, ruminal microbiota transplantation from SARA cows (S-RMT) caused mastitis and aggravated *S*. *aureus*-induced mastitis, while these changes were reversed by HEX. Our findings indicate that HEX effectively attenuates *S*. *aureus*- and SARA-induced mastitis by limiting inflammation and repairing barrier integrity, ultimately highlighting the important role of host or microbiota metabolism in the pathogenesis of mastitis and providing a potential strategy for mastitis prevention.

## Author summary

Although emerging evidence has shown that gut microbiota-derived metabolites regulate pathogen invasions, there is little evidence to show how gut microbiota-derived metabolites influence pathogen invasion in distal organs such as the mammary glands. In this study, untargeted metabolomics was used to assess the metabolic changes in rumen fluid and milk from SARA-associated mastitis cows. We found that SARA-associated mastitis cows had reduced HEX levels compared with healthy cows. Treatment mice with HEX ameliorated *S*. *aureus*-induced mastitis in mice, which was related to suppression of mammary inflammatory responses and repair of the blood-milk barrier. Moreover, HEX inhibited the activation of NF-κB pathway caused by *S*. *aureus* in MMECs, which was involved in the activation of PPARα and subsequent SIRT1. Furthermore, we showed that S-RMT induced mastitis and exacerbated *S*. *aureus*-induced mastitis in mice, while administration mice with HEX reversed these changes. Altogether, our data showed that HEX alleviates *S*. *aureus*- and SARA-induced mastitis by inhibiting inflammatory responses and repairing barrier integrity, which provides not only a better understanding for “gut-mamamry axis”-mediated mastitis pathogenesis, but also implies a potential strategy for the prevention of mastitis and other diseases based on regulation of gut metabolism.

## Introduction

The mammary gland is a specialized organ for the secretion of milk in female animals. Milk is secreted by mammary epithelial cells during lactation. However, the structure of the mammary gland is destroyed and normal milk secretion from alveolar epithelial cells is impaired in the context of mammary diseases including mastitis. Mastitis, a disease that seriously threatens human and animal health and induces tremendous economic losses around the world, is mainly characterized by inflammatory cell infiltration and the destruction of the acinar structure of the mammary gland [1–3]. Multifarious studies have shown that pathogenic microorganism invasion is the major cause of mastitis, including *Staphylococcus aureus* (*S*. *aureus*) [4], *Escherichia coli* [1], and *Streptococcus uberis* [5]. *S*. *aureus* is considered to be one of the most prevalent pathogens of mastitis, and can cause blood-milk barrier leakage characterized by the accumulation of serum albumin in milk and decrease in tight junction (TJ) protein levels [2,6,7]. Although antibiotics have contributed to the elimination of pathogens and serve as the main method of mastitis treatment, they have been insufficient due to increasing concerns regarding drug residues, bacterial resistance, and the role of host factors in mastitis pathogenesis. In addition, bacterial elimination does not improve pathogen-caused disruption of barrier function and the depletion of the commensal microbiota by antibiotics may exacerbate mastitis [7,8], which implies that other factors are involved in the pathogenesis of mastitis. Based on recent studies, endogenous factors, such as rumen microbiota, are also closely related to the occurrence and progression of mastitis [9]. For instance, subacute ruminal acidosis (SARA), a typical case model of rumen microbiota disorder in cows, has been demonstrated to increase the incidence of mastitis [10–12]. Similarly, our recent study revealed that SARA caused rumen microbiota disturbance, increased susceptibility to pathogens in the mammary gland and subsequently promoted the development of mastitis [9]. However, the mechanism whereby SARA promotes mastitis and its underlying molecular mechanism remain to be further explored.

Small molecule metabolites derived from host or microbiota are involved in the regulation of host physiology, immunity and disease outcomes [13]. The well-known metabolites include microbiota-derived short-chain fatty acids (SCFAs) [14], tryptophan-derived ligands of aryl hydrocarbon receptor (AhR) [15], and bile acids [16], which have been reported to participate in multiple diseases. Our previous study revealed that gut dysbiotic mice had reduced SCFAs levels and weakened AhR activation compared with specific pathogen free mice, which developed more serious mastitis upon pathogen invasions, which reversed following compensation of SCFAs and AhR ligands [7,8], demonstrating that beneficial metabolites contribute to the regulation of the outcomes of mastitis. Carol A et al. found that western diet, which is high in fat and low in fiber [17], can significantly alter mammary metabolism and increase inflammatory tendencies [18]. SARA was attributed to the increased consumption of a high-concentrate diet (HCD) in cows, which have been reported to have distinct metabolic profiles in the rumen [19]. Currently, only little is known about the metabolic changes caused by SARA in milk. To explore the role of host or microbiota metabolism in the pathogenesis of SARA-associated mastitis, metabolomics analysis for rumen fluid and milk from healthy cows and SARA-associated mastitis cows was performed in our previous study [9]. Hexadecanamide (HEX) was identified as the main significantly reduced metabolite in the rumen and milk of SARA-associated mastitis cows. HEX is a high melting point amide non-ionic surfactant that exists in many animals and plants, such as the hippocampus nucleus of normal mice, charcoal Kerandang in non-edible parts, garcinia kolaA, hydroalcoholic pork lard, and Ganoderma lucidum extract. Of note, studies related to the metabolic pathway and molecular mechanism of HEX are very scarce. Some previous studies found that HEX has anti-allergic, antioxidant, and neuroprotective effects [20–24]; however, the role of HEX in the pathogenesis of mastitis and the potential mechanism remain unknown.

In the present study, we investigated the role of HEX in a mouse mastitis model caused by infection or ruminal microbial transplantation (RMT) from SARA-associated mastitis cows (S-RMT) [7,9]. HEX was found to alleviate *S*. *aureus*-caused mastitis by depressing inflammatory response and maintaining blood-milk barrier integrity. The underlying mechanism was involved in peroxisome proliferator-activated receptor-α (PPARα)-mediated the activation of sirtuin1 (SIRT1), which blocked NF-κB pathway activation caused by *S*. *aureus*. HEX also ameliorated mammary and colonic inflammation and barrier injury caused by S-RMT. S-RMT aggravated *S*. *aureus*-induced mastitis; however, the severity of mastitis was reversed by HEX via the repair of barrier function and reduction of inflammation. Taken together, we clarified the protective effect of HEX in SARA- and *S*. *aureus*-induced mastitis, which may provide a potential strategy for mastitis prevention and highlight the protective role of beneficial endogenous metabolites in regulating disease outcomes.

## Results

### HEX alleviates *S*. *aureus*-induced mastitis in a dose-dependent manner in mice

**Fig 1:**
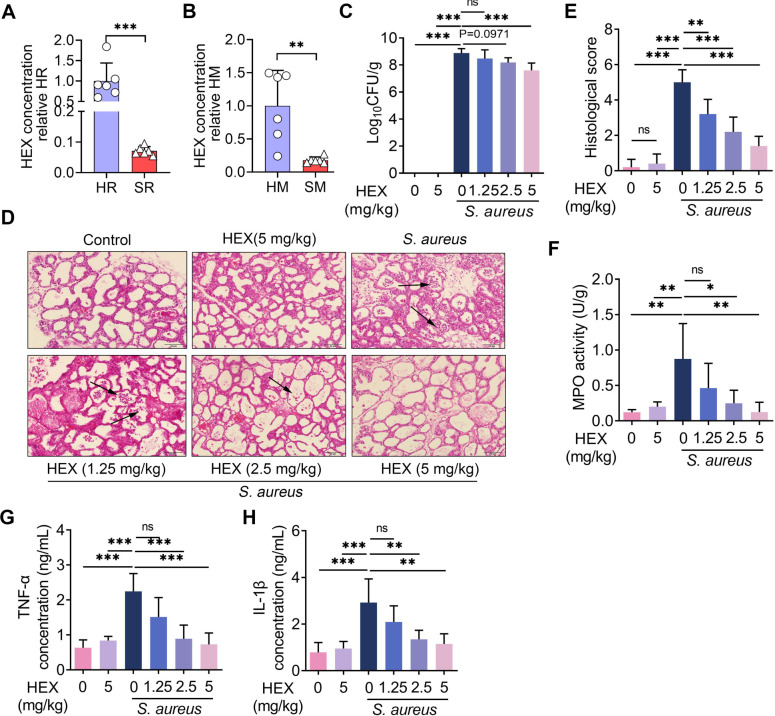
HEX alleviates *S*. *aureus*-induced mastitis. **A-B.** The content of HEX in rumen fluid from healthy cows (HR), cows with SARA-associated mastitis (SR), and in milk from healthy cows (HM) and SARA-associated mastitis cows (SM) was measured (n = 6). **C-H.** Mice were continuously administered HEX (1.25, 2.5, 5 mg/kg) via gavage for 15 days and then infected with *S*. *aureus* (10^8^ CFU/50 μL) for 24 h. The mammary gland tissue was subsequently collected. **C.** The concentration of *S*. *aureus* in the mammary gland was determined by plate coating (n = 5). **D-E.** Representative images of H&E-stained mammary gland (scale bar: 50 μm) and the inflammatory score (n = 5). Black arrows indicate the infiltration of inflammatory cells. **F.** MPO activity of the mammary glands was assessed using the MPO assay kit (n = 5). **G-H.** The levels of TNF-α and IL-1β in the mammary glands from different groups are shown (n = 5). Data are presented as boxplots (A-B) or means ± SD (C and E-H). The Mann-Whitney *U* test (A-B) and one-way ANOVA (C and E-H) were performed for statistical analysis. *p < 0.05, **p < 0.01, and ***p < 0.001 indicate statistical significance.

HEX was found to be reduced in both rumen fluid and milk from cows with SARA-associated mastitis compared to healthy cows **(Fig 1A and 1B)**. To investigate the role of HEX in mastitis, mice were orally administered HEX for 15 consecutive days in the context of *S*. *aureus*-induced mastitis. *S*. *aureus* treatment induced a significant increase in mammary bacterial loads compared with the control, while HEX treatment reduced mammary *S*. *aureus* loads in a dose-dependent manner compared with that induced by *S*. *aureus* treatment **(Fig 1C)**. Consistently, HEX treatment attenuated *S*. *aureus*-induced mammary damage in a dose-dependent manner, based on the reduced inflammatory cell infiltration and structure destruction compared with the *S*. *aureus* treatment **(Fig 1D and 1E)**. Likewise, dose-dependent decreases in several inflammatory markers during mastitis, including MPO activity **(Fig 1F)**, and TNF-α **(Fig 1G)** and IL-1β **(Fig 1H)**, were detected after HEX treatment compared with *S*. *aureus* treatment. Collectively, these results suggest that HEX mitigates *S*. *aureus*-induced mastitis in mice.

### HEX improves *S*. *aureus*-induced blood-milk barrier injury in mice

**Fig 2:**
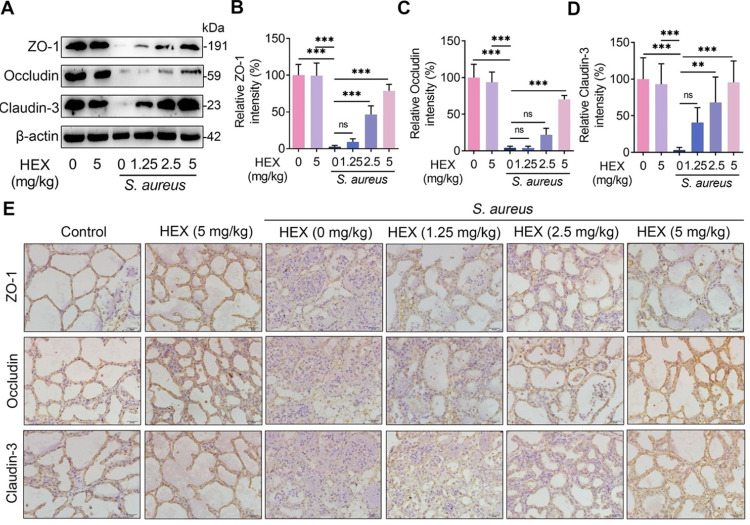
HEX improves *S*. *aureus*-caused disruption of the blood-milk barrier. **A-D.** Western blotting was performed to detect the protein levels of ZO-1, Occludin, and Claudin-3 in the mammary gland (n = 5). **E**. Representative immunohistochemical image of the mammary glands by ZO-1, Occludin, and Claudin-3 antibodies staining (scale bars, 20 μm). Data are expressed as mean ± SD (B-D). ns, no significance, *p < 0.05, **p < 0.01, and ***p < 0.001 indicate significant statistical difference by one-way ANOVA (B-D).

Based on mounting studies, the increase in mammary leukocyte infiltration and inflammation is attributed to the impaired integrity of the blood-milk barrier and the increased permeability [7–9]. Therefore, we investigated whether HEX restores *S*. *aureus*-induced decrease in barrier integrity. Consistent with previous findings, we found that *S*. *aureus* significantly reduced the mammary TJs proteins, ZO-1, Occludin, and Claudin-3 levels compared with the control **(Fig 2A–2E),** suggesting that *S*. *aureus* disrupted the integrity of the blood-milk barrier. Notably, these decreases in the TJs proteins, ZO-1, Occludin, and Claudin-3 caused by *S*. *aureus* were reversed after HEX treatment in a dose-dependent manner **(Fig 2A–2E).** Taken together, these results suggest that HEX repairs the blood-milk barrier injury induced by *S*. *aureus* in mice.

### HEX restores the decrease in TJs proteins and inhibits NF-κB activation caused by *S*. *aureus* in MMECs

**Fig 3:**
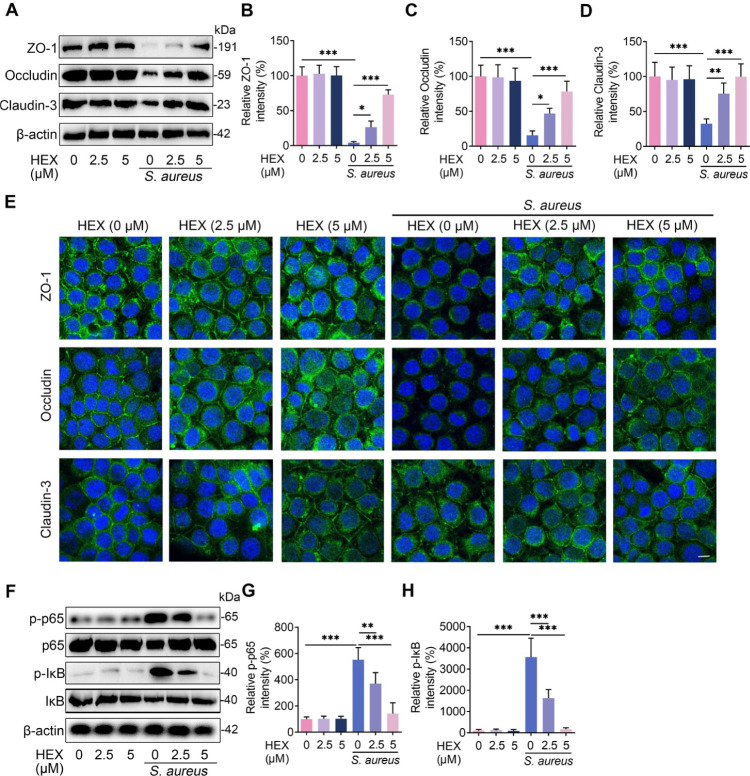
HEX enhances barrier integrity and limits the NF-κB signaling pathway in MMECs. **A-D.** The protein levels of ZO-1, Occludin, and Claudin-3 were assessed after HEX pretreatment (0, 2.5, and 5 μM) for 2 h and *S*. *aureus* treatment (10^5^ CFU) for 24 h (n = 5). **E**. Immunofluorescence staining was performed to detect TJs protein ZO-1, Occludin, and Claudin-3 expressions in MMECs (scale bars, 10 μm). **F-H.** Protein levels in the NF-κB signaling pathway, including IκB, p65, and phosphorylation levels of IκB and p65, were detected with HEX pretreatment (0, 2.5, and 5 μM) for 2 h and *S*. *aureus* treatment (10^5^ CFU) for 24 h (n = 5). One-way ANOVA (B-D and G-H) was used, and values are presented as the mean ± SD (B-D and G-H). *p < 0.05, **p < 0.01, and ***p < 0.001 indicate significantly different from each group. ns, no significance.

To confirm the role of HEX in barrier repair, we treated MMECs with different doses of HEX (0, 2.5 and 5 μM) and then stimulated them with *S*. *aureus*. We did not observe increase in TJs proteins, including ZO-1, Occludin, and Claudin-3, in only HEX treatment groups compared with the control group in MMECs **(Fig 3A–3E)**. However, the decrease in ZO-1, Occludin, and Claudin-3 caused by *S*. *aureus* in MMECs were reversed after HEX treatments in a dose-dependent manner **(Fig 3A–3E)**, which suggests that HEX repairs the barrier integrity by increasing the expression of the TJs proteins via the limiting of inflammatory responses. The NF-κB signaling pathway has been reported to play an important role in inflammatory response and is involved in the pathogenesis of *S*. *aureus*-induced mastitis [7,25]. *S*. *aureus* treatment was found to upregulate the protein levels of p-p65 and p-IκB compared with the control treatment **(Fig 3F–3H)**. In contrast, HEX significantly reversed these increases in a dose-dependent manner in MMECs **(Fig 3F–3H)**. Together, these results indicate that HEX markedly ameliorates the disruption of the epithelial barrier and inhibits the activation of the NF-κB pathway caused by *S*. *aureus* in MMECs.

### Treatment with HEX limits the activation of mammary NF-κB via activating the PPARα-SIRT1 axis

**Fig 4:**
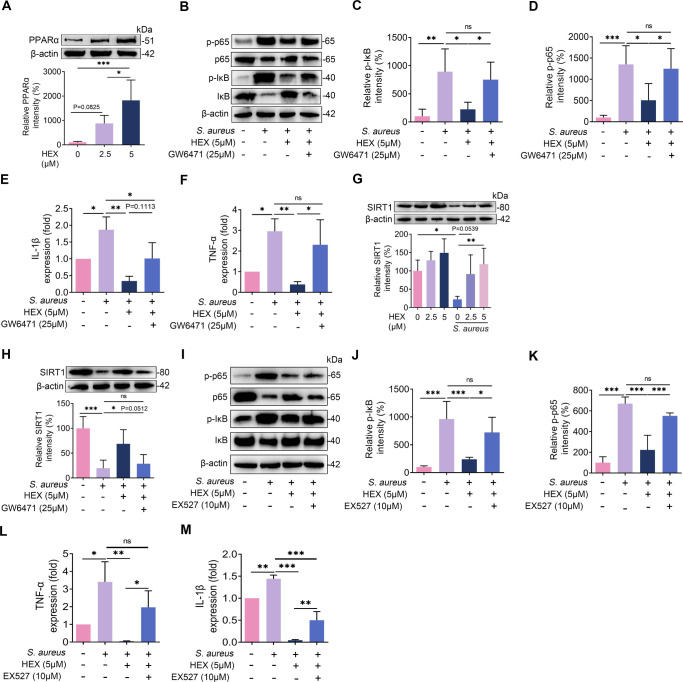
HEX ameliorates *S*. *aureus*-induced inflammation in MMECs through PPARα-SIRT1-NF-κB axis. **A-F and H.** GW6471 (25 μM) and HEX (5 μM) were performed for 2 h followed by *S*. *aureus* (10^5^ CFU) treatment for the next 24 h. **A.** The relative intensities of PPARα was analyzed by western blotting (n = 5). **B-D.** Representative image of IκB, p65, and phosphorylation levels of IκB and p65 were shown by western blotting (n = 5). **E-F.** Inflammatory marker TNF-α and IL-1β were determined using qPCR from different groups (n = 3). **G.** The cells were pretreated with HEX (0, 2.5, and 5 μM) for 2 h and then treated with *S*. *aureus* (10^5^ CFU) for 24 h. The relative level of SIRT1 was measured (n = 5). **H.** The relative intensity of SIRT1 was detected from different groups (n = 5). **I-M.** The cells were pretreated with 10 μM EX527 for 2 h and then stimulated with 10^5^ CFU *S*. *aureus* for 24 h (n = 5). **I-K.** Representative image of IκB, p65, and phosphorylation levels of IκB and p65 from the indicated cells. The levels of IκB, p65, and phosphorylation levels of IκB and p65 were detected (n = 5). **L-M.** The levels of TNF-α (**L**) and IL-1β (**M**) were determined by qPCR (n = 3). Data are presented as the means ± SD (A, C-H and J-M) and one-way ANOVA was performed for statistical analysis (A, C-H and J-M). *p < 0.05, **p < 0.01, and ***p < 0.001 indicate significant difference. ns, no significance.

HEX was found in mice as an endogenous ligand of PPARα [21,26]. We observed that HEX markedly increased PPARα expression in a dose-dependent manner **(Fig 4A)**. To confirm the role of PPARα activation in context of HEX, we blocked PPARα activation with specific inhibitor GW6471 [27]. GW6471 treatment significantly reversed the decrease in p-p65, p-IκB and proinflammatory cytokine TNF-α production (but slightly affected IL-1β) due to HEX treatment in the *S*. *aureus*-treated MMECs **(Fig 4B–4F).** Sirtuin1 (SIRT1), a NAD-dependent deacetylase, regulates the NF-κB signal pathway activation and has potential anti-inflammatory effect in cells [28,29]. We also found that a dose-dependent increase in SIRT1 expression was estimated after HEX treatment relative to *S*. *aureus* treatment **(Fig 4G)**. Similarly, higher level of SIRT1 was tested in the HEX + *S*. *aureus* group compared with the *S*. *aureus* group, but this level was reduced after GW6471 treatment **(Fig 4H)**. Then, we investigated whether SIRT1 is involved in PPARα-mediated NF-κB inhibition by using SIRT1 specific inhibitor EX527. As expected, pretreatment with EX527 reversed the protective effect of HEX on NF-κB activation and proinflammatory cytokine production **(Fig 4I–4M)**. Taken together, these results suggest that HEX ameliorates *S*. *aureus*-induced mastitis by the PPARα-SIRT1-NF-κB axis.

### HEX attenuates SARA-induced systemic inflammation and intestinal injury

**Fig 5:**
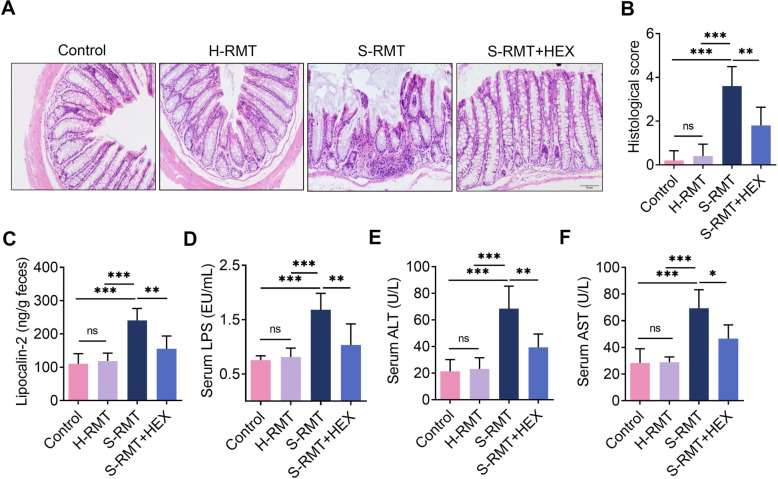
HEX ameliorates SARA-induced systemic inflammation. Mice were administered an oral gavage of rumen fluid from healthy cows, mastitis cows and SARA cows, as well as HEX and PBS for 10 days. **A-B.** Colon tissue sections of mice were stained with H&E (scale bar: 50 μm). **C.** The level of LCN2 in the feces of mice was determined using the LCN2 assay kit (n = 5). **D-F.** LPS assay, and ALT assay, and AST assay were performed to determine the concentration of serum LPS, ALT, and AST, respectively (n = 5). One-way ANOVA (B-F) was performed, and data are presented as mean ± SD (B-F). *p < 0.05, **p < 0.01, and ***p < 0.001 suggest significant difference from each group. ns, no significance.

SARA is a metabolic disorder that promotes rumen and hindgut microbiota dysbiosis, resulting in the translocation of immunogenic compounds into the bloodstream and ultimately systemic inflammation [9]. Considering the role of SARA in mastitis [9] and the decrease in HEX in SARA cows, we investigated whether HEX can alleviate SARA-induced inflammatory responses. S-RMT significantly induced colonic inflammation via leukocyte infiltration and histological injury compared with the control or H-RMT **(Fig 5A and 5B)**. In contrast, HEX effectively reduced colonic inflammation compared with S-RMT **(Fig 5A and 5B)**. Moreover, a higher level of LCN2, an indicator of intestinal inflammation [30], was observed in the S-RMT group compared with the control or H-RMT groups, and this increase in LCN2 was reduced in the HEX treatment group compared with the S-RMT group **(Fig 5C)**. Likewise, S-RMT markedly increased serum LPS level compared with that in the control or H-RMT groups **(Fig 5D)**. However, HEX reduced the serum LPS content compared with the S-RMT **(Fig 5D)**. Higher levels of serum ALT and AST were detected in the S-RMT group relative to the control or H-RMT groups, but these levels were reduced after HEX treatment **(Fig 5E and 5F)**. These results indicate that HEX alleviates S-RMT-induced systemic inflammation and intestinal damage.

**Fig 6:**
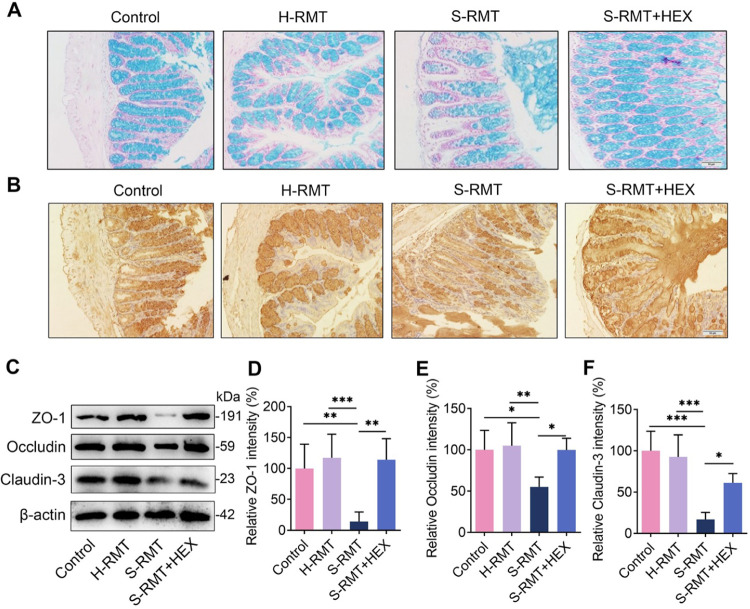
HEX rescues SARA-induced intestinal barrier injury. **A-B.** Representative images of mucin staining of colon tissue sections with damage of the intestinal barrier are shown. **C-F.** The protein levels of TJs, including ZO-1, Occludin, and Claudin-3, were analyzed by western blotting (n = 5). The results are presented as mean ± SD (D-F). ns, no significance, *p < 0.05, **p < 0.01, and ***p < 0.001 indicate statistical significance based on one-way ANOVA (D-F).

Increased serum LPS level was associated with impaired gut barrier integrity [31]. Therefore, we investigated whether S-RMT changed the intestinal integrity regulated by HEX. S-RMT was demonstrated to reduce the number of the goblet cells compared with the control or H-RMT **(Fig 6A),** and this decrease was reversed upon HEX treatment **(Fig 6A).** To confirm these results, colonic mucin-2, a major antimicrobial protein produced by goblet cells that constitutes the first barrier to separate epithelial cells from commensal microbiota [32,33], was determined. Consistently, a lower mucin-2 level was detected in the S-RMT group relative to that in the control or H-RMT groups **(Fig 6B)**; however, HEX increased mucin-2 expression compared with that induced by S-RMT **(Fig 6B)**. These results suggest that HEX improves S-RMT-induced mucosal barrier injury. We proceeded to investigate the influence of RMT and HEX on the colonic epithelial barrier. S-RMT was found to reduce the colonic TJs proteins, ZO-1, Occludin, and Claudin-3, compared with the control or H-RMT **(Fig 6C–6F)**. HEX treatment improved these decreases compared with S-RMT treatment **(Fig 6C–6F)**. Overall, our results suggest that HEX improves S-RMT-induced systemic inflammation and intestinal barrier injury.

### HEX attenuates SARA-induced mastitis and blood-milk barrier injury in mice

**Fig 7:**
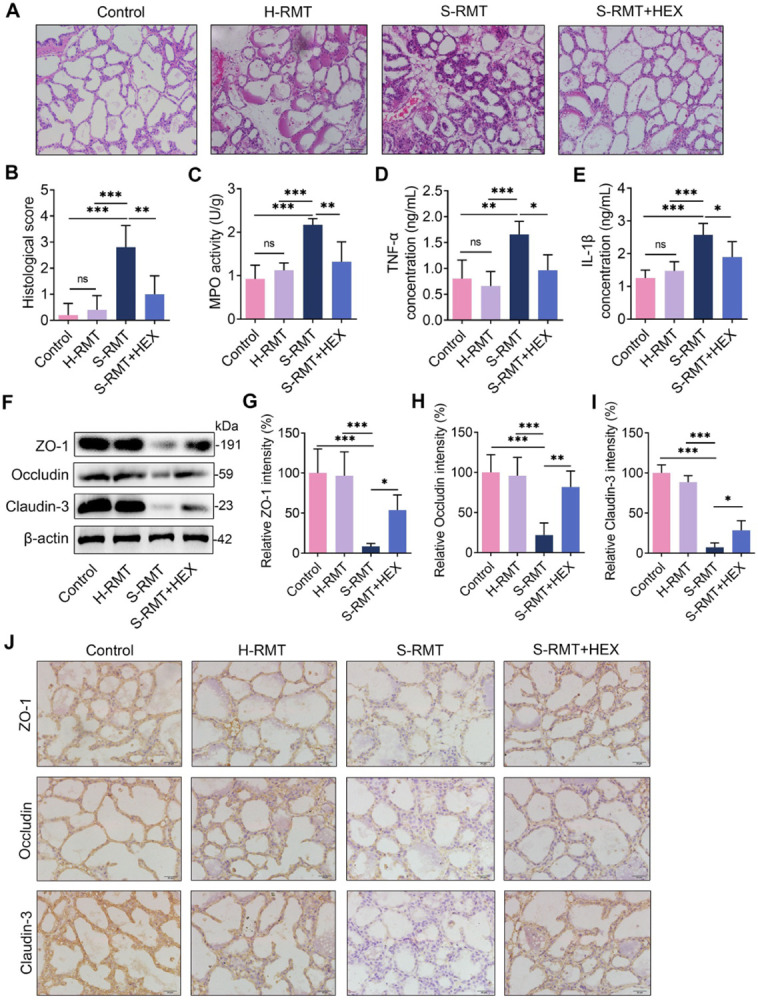
HEX mitigates SARA-induced inflammatory response and blood-milk damage in the mammary glands. PBS, HEX, and rumen fluid from healthy cows, and SARA-associated mastitis cows were administered via gavage to mice for 10 days. Finally, the mammary gland tissues were collected for detection. **A-B.** The pathological changes and inflammatory scores in different groups are shown. **C-E.** MPO activity (**C**), and the levels of TNF-α (**D**) and IL-1β (**E**) were determined (n = 5). **F-I.** ZO-1, Occludin, and Claudin-3 protein levels and intensity analysis were performed (n = 5). **J**. Representative immunohistochemical image of the mammary glands by ZO-1, Occludin, and Claudin-3 antibodies staining (scale bars, 20 μm). One-way ANOVA (B-E and G-I) was performed and values are presented as mean ± SD (B-E and G-I). *p < 0.05, **p < 0.01, and ***p < 0.001 indicate marked difference from each group. ns, no significance.

We next investigated the role of HEX in SARA-associated mastitis. S-RMT was found to induce obvious mammary injury compared with the control or H-RMT **(Fig 7A and 7B)**, while HEX significantly reversed S-RMT induced mammary injury **(Fig 7A and 7B)**. Likewise, S-RMT markedly increased the mammary inflammatory markers, including MPO activity, and TNF-α and IL-1β contents, compared with the control or H-RMT **(Fig 7C–7E)**. HEX treatment distinctly mitigated the upregulation of the inflammatory markers caused by S-RMT **(Fig 7C–7E).** Consistently, mice in the S-RMT group had reduced levels of the TJs proteins, ZO-1, Occludin, and Claudin-3, compared with those in the control or H-RMT groups (**Fig 7F–7J**), and these decreases were reversed by HEX compared with the S-RMT (**Fig 7F–7J**). These results suggest that HEX alleviates mastitis and repairs the blood-milk barrier caused by SARA.

### SARA aggravates *S*. *aureus*-induced mastitis but is reversed by HEX in mice

**Fig 8:**
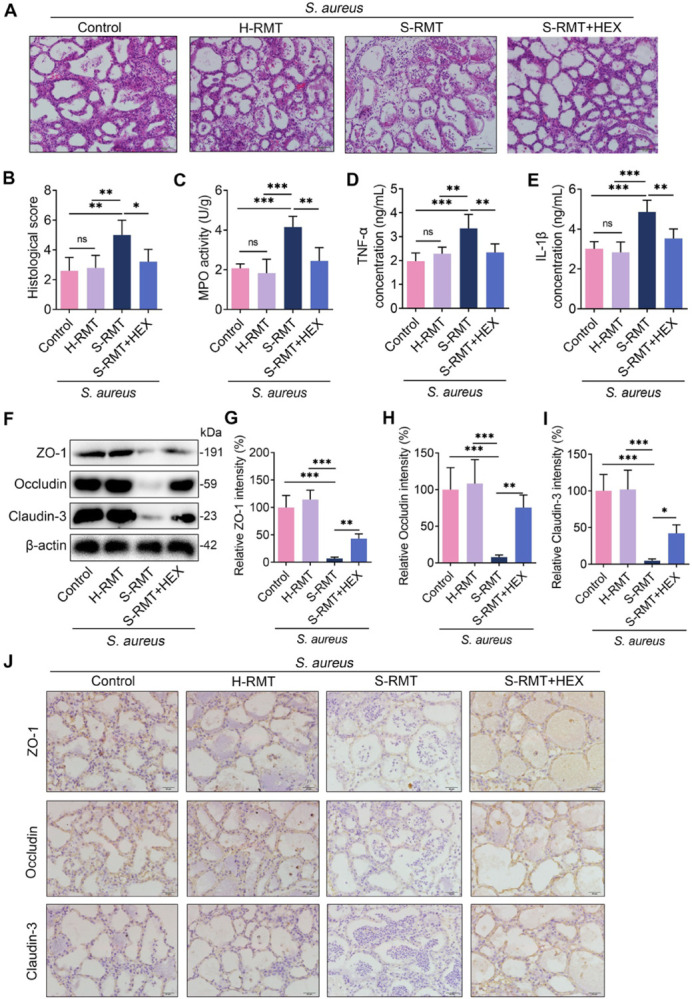
S-RMT facilitates *S*. *aureus*-induced mastitis and is reversed by HEX. PBS, HEX, and rumen fluid from healthy cows, SARA-associated mastitis cows were administered via gavage to mice for 10 days, followed by *S*. *aureus* (10^8^ CFU/50 μL) treatment for 24 h. **A-B.** Mammary sections were stained with H&E after S-RMT and *S*. *aureus* treatment; H&E was used to assess the degree of inflammatory damage. **C-E.** The concentrations of proinflammatory factors, including TNF-α and IL-1β, and the MPO activity were measured (n = 5). **F-I.** The content of TJs after S-RMT and *S*. *aureus* treatment was determined by western blotting (n = 5). **J**. Representative immunohistochemical image of the mammary glands by ZO-1, Occludin, and Claudin-3 antibodies staining (scale bars, 20 μm). One-way ANOVA (B-E and G-I) was applied for statistical analysis. Data are presented as mean ± SD (B-E and G-I). *p < 0.05, **p < 0.01, and ***p < 0.001 indicate marked difference from each group. ns, no significance.

In our previous study, SARA cows were found to have increased susceptibility to *S*. *aureus*-induced mastitis [9], and gut dysbiotic mice developed more serious mastitis caused by *S*. *aureus* [7]. We next investigated the influence of S-RMT and the effect of HEX on *S*. *aureus*-induced mastitis in mice. S-RMT mice displayed a greater degree of mammary damage compared with control or H-RMT mice upon *S*. *aureus* infection **(Fig 8A and 8B)**. Notably, HEX treatment reduced S-RMT-facilitated mammary injury caused by *S*. *aureus* **(Fig 8A and 8B)**. Moreover, higher levels of mammary inflammatory markers, including MPO activity, and TNF-α and IL-1β contents, were detected in mice in the S-RMT + *S*. *aureus* group relative to those in the *S*. *aureus* or H-RMT **+** *S*. *aureus* groups **(Fig 8C–8E)**. These increases in mammary proinflammatory markers were reduced after HEX treatment compared with S-RMT + *S*. *aureus* treatment **(Fig 8C–8E).** Mice in the S-RMT + *S*. *aureus* group had reduced mammary TJs protein levels compared with those in the *S*. *aureus* or H-RMT + *S*. *aureus* groups **(Fig 8F–8J)**; these changes were reversed by HEX treatment **(Fig 8F–8J)**. Collectively, our results suggest that SARA aggravates *S*. *aureus*-induced mastitis, but is reversed by HEX treatment in mice.

## Discussion

Mastitis, a widespread disease in dairy cows, is thought to be an inflammatory response of the mammary gland caused by environmental pathogens [34]. However, in recent years, multiple crucial causative factors of mastitis have been identified, such as rumen microbiota [9]. SARA, a typical case that causes changes in rumen microbial composition, was employed to assess the interaction between rumen microbiota and mastitis [9]. SARA promoted the pathogenesis of mastitis by triggering systemic low-grade inflammation, leading to LPS accumulation and inflammation in the mammary gland [9]. Meanwhile, significant changes in HEX were found in rumen fluid and the milk of cows with SARA-associated mastitis; however, the role of HEX in SARA-associated mastitis and the underlying mechanisms are poorly understood. In this study, HEX effectively ameliorated *S*. *aureus*-caused mastitis in mice. The underlying mechanism was involved in the limitation of the NF-κB axis by the PPARα-SIRT1 pathways and increased TJs protein levels in a dose-dependent manner. S-RMT, and not H-RMT, significantly replicated the mastitis model and caused systemic damage in mice; however, these changes were reversed by HEX. Notably, S-RMT aggravated *S*. *aureus*-induced mastitis symptoms and HEX acts as a palliative in the process. Collectively, HEX is a potential prophylactic agent for SARA- and *S*. *aureus*-induced mastitis in mice.

The undamaged blood-milk barrier not only ensures effective nutrient exchange between milk and blood, but also acts as an immune barrier to resist the invasion of pathogens. However, when the barrier function is destroyed, the progression of mastitis is promoted [2,35,36]. Consistent with the findings of previous studies, *S*. *aureus* was found to disrupt the integrity of the blood-milk barrier characterized by increasing albumin in the acinar lumen, which might be a marker of increased blood-milk barrier permeability [7,37]. Interestingly, HEX pretreatment recovered the barrier function and improved mastitis. Barrier function is dominated by junctional complexes, such as cell-to-cell TJs, adhesive junctions, desmosomes, and gap junctions [38]. TJs, which are mainly composed of two transmembrane spanning structural proteins, Occludin and Claudin, that are linked to the actin cytoskeleton by scaffolding proteins, such as ZO-1 [39,40], are among these junctional complexes. Here, we found that *S*. *aureus* downregulated the expression levels of TJs [41], including ZO-1, Occludin, and Claudin-3; however, HEX reversed these changes, thereby demonstrating its protective role in barrier functions.

MPO activity is a marker of the activation of neutrophils, which directly reflects the level of neutrophil aggregation in the mammary glands [42,43]. Herein, HEX administration mitigated MPO activity induced by *S*. *aureus*. Proinflammatory cytokines are involved in the pathological process of mastitis, mainly including TNF-α, IL-1β, and IL-6 [2,44], which are associated with the NF-κB pathway that regulates the production of cytokines [45] and is involved in mastitis pathogenesis [7,8]. After exposure to *S*. *aureus*, increased phosphorylation of p65 and IκB, a marker of activation of the NF-κB axis, was observed. Further, the transcription factor, NF-κB, was transferred from the cytoplasm to the nucleus, thereby triggering the expression of TNF and IL-1 [4,46]. However, HEX depressed the activation of NF-κB pathway and PPARα was identified as an endogenous receptor for HEX [26]. Then, we found that PPARα was increased in a dose-dependent manner after HEX treatment. In turn, GW6471, a typical inhibitor of PPARα, significantly reversed the effect of HEX on NF-κB pathway and subsequent proinflammatory cytokines in MMECs. Moreover, activation of PPARα upregulated expression and activity of SIRT1 [47]. Blocking SIRT1 with EX527 impaired the protective effect of HEX. Collectively, HEX alleviated inflammatory response via PPARα-SIRT1-NF-κB axis *in vitro* and HEX also rescued *S*. *aureus*-induced mastitis in a dose-dependent manner *in vivo*.

SARA is often caused by long-term feeding of a HCD, and a decline in ruminal pH and rumen microbial imbalance [48]. Once the host develops SARA, increased ruminal and intestinal epithelial permeability will ensure, allowing harmful products, such as LPS, into the blood circulation that lead to systemic inflammation [9]. During the lactation period, the acceleration of blood circulation leads to the accumulation of LPS in the mammary gland and exacerbates the inflammatory response induced by pathogens [9]. Consistent with previous findings [9,49], mice in the S-RMT group developed a greater degree of inflammatory response and blood-milk barrier leakage. Upon *S*. *aureus* infection, S-RMT mice also displayed more serious inflammatory responses. However, HEX alleviated these pathological changes, suggesting the protective role of HEX and highlighting that host metabolism significantly regulates disease development. The liver is a vital organ for removing toxins and maintaining metabolic homeostasis. Thus, to some extent, the immune status of the liver reflects the health of the host [50]. Once LPS enters the liver through the bloodstream, the hepatocytes are injured due to the accumulation of LPS in the liver, which in turn leads to an increase in the clearance of LPS in the liver; however, the capacity of the removal is distinctly reduced [51]. Although SARA caused significant upregulation of LPS levels in serum, HEX reversed this change. Meanwhile, ALT and AST that are important indicators reflecting liver function, were markedly decreased in the S-RMT + HEX group compared to the S-RMT group. HEX ameliorated the increase in intestinal barrier permeability caused by S-RMT, which agrees with the notion that S-RMT could disrupt the intestinal barrier [19] and suggests the important role of HEX.

Overall, our findings suggest that HEX ameliorates SARA-associated and *S*. *aureus*-induced mastitis by inhibiting the inflammatory response and regulating blood-milk barrier integrity in mice. To our knowledge, this is the first study to demonstrate that HEX plays a protective role in mastitis caused by dysbiosis and infection, thereby serving as a reference for the prevention of mastitis and other infectious and metabolic diseases.

## Materials and methods

### Ethics statement

The study protocol was approved by the Ethics Committee on the Use and Care of Animals at Jilin University (Changchun, China) (approval number: pzpx20201023022). The full proposal was considered by the IACUC ethics committee, which approved the animal care and use permit license.

### Materials

HEX (Y40637) was purchased from Yuanye biotechnology Co., Ltd, (Shanghai, China). GW6471(880635-03-0) was bought from Rhawn (Shanghai, China). EX527 (abs812154) was obtained from Absin (Shanghai, China). The primary antibodies, including β-actin (1:1000; #AF7018; RRID: AB_2839420), p65 (1:1000; #AF5006; RRID: AB_2834847), IκB (1:1000; #AF5002; RRID: AB_2834792), phosphorylation-p65 (p-p65) (1:1000; #AF2006; RRID: AB_2834435) and IκB (p-IκB) (1:1000; #AF2002; RRID: AB_2834433), ZO-1 (1:200 or 1:1000; #AF5145; RRID: AB_2837631), Occludin (1:200 or 1:1000; #DF7504; RRID: AB_2841004), Claudin-3 (1:200 or 1:1000; #AF0129; RRID: AB_2833313) and mucin (1:200; #DF8390; RRID: AB_2827875), were obtained from Affinity Biosciences (OH, USA). RRARα (1:1000; bs-23398R) and SIRT1 (1:1000; bs-2257R) were obtained from Bioss (Beijing, China). Ampicillin (Cat# A5354), neomycin (Cat# N6386), metronidazole (Cat# 16677) and vancomycin (Cat# V2002) were obtained from Sigma (St. Louis, MO, USA,). Tumor necrosis factor (TNF)-α (Cat. No. 430915) and interleukin (IL)-1β (Cat. No.432615) enzyme-linked immunosorbent assay (ELISA) kits were purchased from Biolegend (San Diego, California, USA). The myeloperoxidase (MPO) (A044-1-1), alanine aminotransferase (ALT) (C009-2-1) and aspartate transaminase (AST) assay kits (C010-2-1) were obtained from Nanjing Jiancheng Bioengineering Institute (Nanjing, China,). The Lipocalin-2 (LCN2) (hj-C1224) and lipopolysaccharide (LPS) assay kits (hj-C1253) were gained from Lanpai biotechnology Co., Ltd (Shanghai, China,). The Alcian blue staining kit (G1560; CAS: 75881-23-1) and Hoechst33342 (C0031) were bought from Solarbio (Beijing, China). The FastStart Universal SYBR Green Master Mix (ROX; 4913914001) was bought from Roche (Switzerland, Basel). Trizol (MF034-1) was gained from Invitrogen (Carlsbad, CA, USA).

### Cows and treatment

Twelve healthy Holstein cows (4–6 years, averaging ~600 kg of weight) were obtained from a farm in Qingzhou, Shandong Province, China, and were none of diseases and drugs treatment within six months. The cows were randomly divided into healthy group and SARA group. SARA was diagnosed by the ruminal PH and mastitis was diagnosed by Somatic cell count (SCC) as previously [52]. The healthy cows were fed a standard diet (grass-leguminous hay), and the SARA cows were fed a high-concentrate diet (70% grain diet). All cows are given adequate water and fodders to meet their daily nutrient requirements. After 8 weeks of treatment, milk and rumen fluid were collected and stored in liquid nitrogen until the metabolomics analysis.

### Untargeted metabolomics

The milk samples (100 mL) were vortex mixed with prechilled methanol (400 μL). Subsequently, the samples were incubated on ice for 5 min and centrifuged at 15000 × g and 4°C for 5 min. The supernatant was diluted with LC-MS grade water to a final concentration of 53% methanol. The samples were then transferred to fresh Eppendorf tubes, which were then centrifuged at 15000 × g and 4°C for 10 min. Finally, the supernatant was injected into the LC-MS/MS system for analysis. The KEGG database (http://www.genome.jp/kegg/) and Lipidmaps database (http://www.lipidmaps.org/) were used for these metabolites analysis.

### Mouse and treatment

The animals were randomly divided into groups via group randomization to ensure that each experiment had an equal sample size at all time points. In all experiments, investigators were group-blinded for all parameters including all data acquisition, sample processing, and data analysis. The number of animals and group sizes were calculated via a priori power analysis using G*Power software (version 3.1, Universität Kiel, Kiel, Germany) and indicated in the figure legends. The effect size *f*, as defined by Cohen (1988), was determined using the population mean, α (error probability) was 0.05 and power (1-β error probability) was set to 0.8. In this experiment, we opted to minimize the number of animals used and attempted to minimize the suffering of animals. Male and female BALB/c mice (21–25 g, 6–8 weeks old) were purchased from Liaoning Changsheng biotechnology Co., Ltd (Benxi, China). Mice were kept in a standard environment with adequate feed and water supplied in individually ventilated cages of the same size, with wood shaving as the bedding material (one male and three females per cage). All mice were housed in a specific pathogen-free facility with a 12-hour light and dark cycle. The temperature and humidity of the environment were maintained at 22 ± 3°C and 35 ± 5%, respectively.

Male mice and female mice were housed together in cages until the female mice became pregnant. Thereafter, the pregnant mice were selected for subsequent experiments. A total of 70 mice were used in this experiment. HEX was dissolved in 0.1% sodium carboxymethyl cellulose at 50°C (0.17, 0.3 and 0.6g/mL). To investigate the effect of HEX on *S*. *aureus*-induced mastitis, mice were randomly divided into six groups (n = 5 per group): Control (0.1% sodium carboxymethyl cellulose treatment), HEX (5 mg/kg), *S*. *aureus* (10^8^ CFU), 1.25 mg/kg HEX + *S*. *aureus*, 2.5 mg/kg HEX + *S*. *aureus*, and 5 mg/kg HEX + *S*. *aureus*. Sodium carboxymethyl cellulose and HEX were administered to mice via oral gavage for consecutive 15 days (once a day) until *S*. *aureus* treatment. The mouse mastitis model was established as previously described [53]. In brief, mice (5–7 days after parturition) were separated from the offspring for 3 h and then were anesthetized with 10% urethane solution (10 mL/kg) via intraperitoneal injection. The nipples and skins around the mammary gland were disinfected with 75% alcohol. Further, the nipples of the fourth pair of mammary glands were gently clamped and exposed *S*. *aureus* (10^8^ CFU/50 μL) was injected via the mammary gland ducts using a 100-μL syringes with a 30-gauge blunt needle. The control group was similarly injected with an equal volume of PBS. Twenty-four hours later, mice were killed, and the mammary gland tissues were collected and coded. The harvested samples were placed at -80°C until analysis.

To determine the protective role of HEX in mastitis caused by SARA, 0.5 g of ruminal content from each cow in the healthy group (n = 6) and SARA group (n = 6) was weighed, and the contents from the same cow group were mixed together as the same donor. Mixed ruminal contents were homogenized and centrifuged (1000 g × 2 min × 4°C), and then supernatants were collected. Mice were randomly divided into eight groups (n = 5 per group), including Control group (each mouse was treated with 200 μL PBS), H-RMT group (each mouse was treated with 200 μL rumen fluid from healthy cows), S-RMT group (each mouse was treated with 200 μL rumen fluid from SARA cows), S-RMT + HEX group (each mouse was treated with 200 μL rumen fluid from cows with SARA and 5 mg/kg HEX); *S*. *aureus* group (each mouse was treated with 200 μL PBS followed by 10^8^ CFU *S*. *aureus*), H-RMT + *S*. *aureus* group (each mouse was treated with 200 μL rumen fluid from healthy cows followed by 10^8^ CFU *S*. *aureus*), S-RMT + *S*. *aureus* group (each mouse was treated with 200 μL rumen fluid from cows with SARA and 10^8^ CFU *S*. *aureus*), S-RMT + HEX + *S*. *aureus* group (each mouse was treated with 200 μL rumen fluid from cows with SARA, 5 mg/kg HEX and 10^8^ CFU S. *aureus*). RMT was performed as previously described [19,31,54]. Briefly, mice were given a cocktail of antibiotics (200 mg/kg ampicillin, neomycin and metronidazole and 100 mg/kg vancomycin) for five consecutive days to eradicate commensal microbiota. After removal of the antibiotics with water for one day, 200 μL of rumen fluid from different groups or PBS was orally administered for three consecutive days followed by once every two days for three weeks [31]. For HEX supplementation, HEX (5 mg/kg) was orally administered after each RMT. Twenty-four hours after the last RMT or HEX treatment, mice were employed to establish the *S*. *aureus*-induced mastitis model as described above.

### Histological analysis

Histological scores based on hematoxylin and eosin (H&E) staining was used to evaluate the severity of injury in the mammary gland and colon in *S*. *aureus*- and SARA-induced mouse model. In brief, the mammary and colonic tissues used for histological analysis were treated with 4% paraformaldehyde for more than 48 h, and then embedded in paraffin to prepare 4 μm sections. The prepared paraffin sections were dewaxed with xylene and different concentrations of alcohol and stained with H&E. Histopathological changes were analyzed via optical microscopy (Olympus, Tokyo, Japan) as previously described [7,8,55] and was presented in **S1 Table**.

### MPO activity assay

The content of neutrophils in mammary glands was determined using an MPO assay kit. In brief, 10% mammary gland tissue homogenate was prepared and the MPO activity was calculated at OD 460 nm according to the manufacturer’s instructions (Nanjing Jianchen, China).

### Proinflammatory cytokine determination

To assess the proinflammatory cytokine levels in mammary glands, 10% tissue homogenate was prepared using PBS and centrifuged at 12000 × rpm for 10 min. Then, the supernatants were collected and the levels of TNF-α and IL-1β were measured by ELISA according to the manufacturer’s instructions (Biolengend, USA).

### Serum LPS, ALT and AST assays

To measure serum LPS, ALT and AST levels, whole blood samples were collected and centrifuged (3000 g × 10 min × 4°C), and serum was harvested for subsequent experiments. Serum LPS was measured using an LPS Assay kit according to the manufacturer’s instruction (Shanghai lanpai, China). Serum ALT and AST were detected using ALT and AST Assay kits according to the manufacturer’s instruction (Nanjing Jianchen, China).

### Fecal LCN2 assay

Fecal LCN2 level was determined using an LCN2 Assay kit according to the manufacturer’s instruction (Shanghai lanpai, China). In brief, fecal samples were collected from the treated mice and used to prepare a 10% homogenate. After centrifugation at 3000 g for 10 min at 4°C, the supernatants were collected for detection.

### Bacteria cultures

*S*. *aureus* (ATCC35556) was purchased from American Type Culture Collection (ATCC, USA). *S*. *aureus* was cultured in TSB medium (Qingdao Haibo, Qingdao, China, HB4114) at 37°C 180 r/min for 8 h to reach the mid-log phase.

### Mammary bacterial burden assay

To evaluate mammary *S*. *aureus* load in mice from different treatment groups, mammary tissues were collected and weighed under sterile conditions. Mammary tissues were prepared for 10% tissue homogenate with PBS and 50 μL tissue homogenate was coated on the Mannitol Salt Agar plates, a selective medium for *S*. *aureus*. Twenty-four hours after incubation, the CFUs on the plates were counted and the *S*. *aureus* load was calculated. All sterile instruments were used in this experiment.

### Alcian blue staining

Colon tissues were divided into 4 μm slices as mentioned above. After dewaxing in xylene and a series of graded alcohols, the colonic sections were stained with an Alcian blue staining assay kit (Solarbio, China, G1560; CAS: 75881-23-1) according to the manufacturer’s instruction.

### Immunochemistry

Immunohistochemistry was used to determine the role of HEX in SARA-induced intestinal injury in mice. The fixed colon sections were dewaxed and rehydrated as mentioned above. Using heat-induced epitope repair techniques, colon slices were boiled in citrate buffer solution (pH 6.0). The prepared sections were treated with endogenous peroxidase blocker (SAP (Mouse/rabbit) IHC kit, MXB, China, KIT-7710) at room temperature for 40 min. After three washes with PBS for 5 min each, the sections were treated with nonimmunized goat serum (SAP (Mouse/rabbit) IHC kit, MXB, China, KIT-7710) for 40 min at room temperature and incubated with ZO-1 (1:200; #AF5145; RRID: AB_2837631, Affinity Biosciences, Beijing, China), Occludin (1:200; #DF7504; RRID: AB_2841004, Affinity Biosciences, Beijing, China), Claudin-3 (1:200; #AF0129; RRID: AB_2833313, Affinity Biosciences, Beijing, China) and mucin antibodies (1:200, #DF8390; RRID: AB_2827875, Affinity Biosciences, Beijing, China) at 4°C overnight. The sections were then incubated with secondary antibodies (goat anti-rabbit IgG) (SAP (Mouse/rabbit) IHC kit, MXB, China, KIT-7710) at room temperature for 30 min and washed three times with PBS every 5 min. The slices were processed and incubated with horseradish peroxidase (HRP) (SAP (Mouse/rabbit) IHC kit, MXB, China) for 20 min at room temperature. After three washes with PBS for 5 min each, color development was achieved using a color developing agent (SAP (Mouse/rabbit) IHC Kit, MXB, China) for 3 min observed under a microscope; color development was terminated by the addition of water. The nuclei were stained with hematoxylin for 5 min, differentiated with 1% muriatic acid alcohol (75%), and treated with ammonium hydroxide for 3 s. After dehydration, the slices were fixed with neutral resin and detected under an optical microscopy (Olympus, Tokyo, Japan).

### Cell culture and treatment

MMECs (HC11 cells) were obtained from the American Type Culture Collection (ATCC, CRL-3062) and cultured in Dulbecco’s modified Eagle’s medium (DMEM) (Hyclone, USA, SH30022.01) supplemented with 10% fetal bovine serum (FBS) (BI, USA, S9040) and 1% penicillin and streptomycin (Hyclone, USA, SV30010) at 37°C in a humidified with 5% CO_2_. For HEX treatment experiment, MMECs (10^6^ cell/mL) were cultured in 6-well plates pretreated with HEX (0, 2.5, 5 μM) for 2 h followed by *S*. *aureus* treatment (10^5^ CFU) for 24 h. For GW6471 treatment experiment, the prepared cells were pretreated with GW6471 (25 μM) and HEX for 2 h prior to *S*. *aureus*. For EX527 treatment experiment, EX527 (10 μM) and HEX was performed 2 h prior to *S*. *aureus* treatment. For all experiments, 10^5^ CFU *S*. *aureus* was added after indicated pretreatment for 24 h. Finally, the cells were collected for subsequent experiments.

### Immunofluorescence staining

Immunofluorescence staining was performed to investigate TJs protein expressions in MMECs. Cells were incubated into 24-well plates prepositioned with polylysine-coated coverslips and cultured for 24h. The cells were then treated with HEX (0, 2.5, 5 μM) and *S*. *aureus* as mentioned above. Next, the cells were fixed with 4% paraformaldehyde for 30 min and blocked with 5% bovine serum albumin (BSA) for 30 min after washing in PBS for three times. Furthermore, the cells were incubated with ZO-1 (1:200; #AF5145; RRID: AB_2837631, Affinity Biosciences, Beijing, China), Occludin (1:200; #DF7504; RRID: AB_2841004, Affinity Biosciences, Beijing, China) and Claudin-3 (1:200; #AF0129; RRID: AB_2833313, Affinity Biosciences, Beijing, China) for 2 h at 37°C. Fluor488-conjugated goat anti-rabbit IgG (#S0018; RRID: AB_2846215; Affinity Biosciences, Beijing, China) were then performed for 30 min at 37°C after washing in PBS for 3 times. The nucleus was stained with Hoechst33342 (Solarbio, Beijing, China, C0031) and finally observed using a confocal microscope (Olympus, Tokyo, Japan).

### Western immunoblotting

Proteins from mammary glands, colons, and MMECs were extracted using tissue protein-extracted reagent (Thermo Fisher Scientific, USA, 78510), and total protein concentration was determined using a BCA Protein Assay Kit (Thermo Fisher Scientific, USA, 23227). Protein samples (30 μg) were separated via sodium dodecyl sulfate-polyacrylamide gel electrophoresis (SDS-PAGE) on a 12% gel, and then transferred to a polyvinylidene fluoride (PVDF) membrane (Millipole, USA, IFVH00010). The prepared PVDF membranes were blocked using 5% skim milk for 3 h and then incubated with specific primary antibodies at 4°C overnight. After 3 washes with TBS-T, these membranes were incubated with HRP-conjugated anti-rabbit IgG (1:20000; Immunoway, USA, RS0002) or HRP-conjugated anti-mouse IgG (1:20000; Immunoway, USA, RS0001) at room temperature for 2 h. Finally, the proteins were visualized using an enhanced chemiluminescence solution (1:1; Yamei, Shanghai, China, SQ202) and the membranes were detected with an ECL Plus Western blotting detection system (Tanon 4500, Shanghai, China) after 3 washes. All western blots were analyzed using Image-Pro Plus 6.0 (Media Cybernetics). β-actin was used as an internal reference protein in this experiment. The western blots are representative of five independent and consistent experiments.

### RNA extraction and qPCR

Cells samples were collected and total RNA was extracted by Trizol (Invitrogen, Carlsbad, CA, USA). In brief, the cells were treated with 1 ml Trizol, followed by chloroform, isopropyl alcohol and 75% anhydrous ethanol and RNase-free water for RNA extraction. cDNA was reverse transcribed by TransStart Tip Green qPCR SuperMix (TransGen Biotech, Beijing, China) and reacted with specific primers using FastStart Universal SYBR Green Master Mix (ROX; Roche, Switzerland, Basel) in a Step One Plus apparatus (Applied Biosystems, Foster City, CA, USA). The specific primers used were shown in **S2 Table.** The reaction conditions were 52°C for 2 min, 95°C for 10 min, 95°C for 15 s and 60°C for 1 min for 40 cycles. The 2^−ΔΔCt^ method was used to calculate the relative expression of genes and GAPDH served as the calibrator.

### Statistical analysis

Group size represents the number of independent values (one data represents per animal, slice, or cell) and all statistical analyses were performed using such independent values (technical replicates were not considered independent values). Statistical analysis was only performed for studies with a group size of at least n = 5 or n = 3. No data points were excluded from the analysis. All data were analyzed using GraphPad Prism version 8.0 (Manufacturer, La Jolla, CA, USA). Data are presented as mean ± SD or boxplots. Comparisons of more than two groups were performed using one-way analysis of variance (ANOVA) and Mann-Whitney *U* test followed by Tukey’s test. For all one-way ANOVAs, Mann-Whitney *U* test, *post hoc* tests were only performed if F achieved *p* < 0.05 and no significant variance existed in homogeneity. Statistical significance was indicated by *p* < 0.05.

## Supporting information

S1 Table Inflammatory scoring criteria of mammary gland tissues. (DOCX)

S2 Table Primers used in this study. (DOCX)

S1 Data Excel spreadsheet containing, in separate sheets, the data points presented in Figs 1–8. (XLSX)
