# Supplementary material for: Hexadecanamide alleviates Staphylococcus aureus-induced mastitis in mice by inhibiting inflammatory responses and restoring blood-milk barrier integrity
Source: PLoS Pathog. 2023 Nov 10;19(11):e1011764. doi: 10.1371/journal.ppat.1011764 (PMC10664928; doi:10.1371/journal.ppat.1011764)
Supplement: S1 Table — (DOCX) [file ppat.1011764.s001.docx]

Table 1 Inflammatory scoring criteria of mammary gland tissues

| Feature | Description | Score |
| --- | --- | --- |
| Hyperemia/Edema | Normal | 0 |
|  | Mild | 1 |
|  | severe | 3 |
| Milk stasis/Acinar necrosis | Normal | 0 |
|  | Milk | 1 |
|  | Moderate | 2 |
|  | Severe | 3 |
| Infiltration with neutrophil | 0-1 Acinar or mammary gland neutrophil | 0 |
|  | 2-5 Acinar or mammary gland neutrophil | 1 |
|  | 6-10 Acinar or mammary gland neutrophil | 2 |
|  | 11-15 Acinar or mammary gland neutrophil | 3 |
|  | 16-20 Acinar or mammary gland neutrophil | 4 |
|  | > 20 Acinar or mammary gland neutrophil | 5 |

Table 2 Primers used in this study

| Gene | Primer | Sequence (5’-3’) |  |
| --- | --- | --- | --- |
| GAPDH | sense | AGGTCGGTGTGAACGGATTTG |  |
|  | antisense | TGTAGACCATGTAGTTGAGGTCA |  |
| TNF-α | sense | CCCTCACACTCAGATCATCTTCT |  |
|  | antisense | GCTACGACGTGGGCTACAG |  |
| IL-1β | sense | GCAACTGTTCCTGAACTCAACT |  |
|  | antisense | ATCTTTTGGGGTCCGTCAACT |  |
