# Supplementary material for: Hexadecanamide alleviates Staphylococcus aureus-induced mastitis in mice by inhibiting inflammatory responses and restoring blood-milk barrier integrity
Source: PLoS Pathog. 2023 Nov 10;19(11):e1011764. doi: 10.1371/journal.ppat.1011764 (PMC10664928; doi:10.1371/journal.ppat.1011764)
Supplement: S2 Table — (DOCX) [file ppat.1011764.s002.docx]

**S2 Table** Primers used in this study

| Gene | Primer | Sequence (5’-3’) |  |
| --- | --- | --- | --- |
| GAPDH | sense | AGGTCGGTGTGAACGGATTTG |  |
|  | antisense | TGTAGACCATGTAGTTGAGGTCA |  |
| TNF-α | sense | CCCTCACACTCAGATCATCTTCT |  |
|  | antisense | GCTACGACGTGGGCTACAG |  |
| IL-1β | sense | GCAACTGTTCCTGAACTCAACT |  |
|  | antisense | ATCTTTTGGGGTCCGTCAACT |  |
